# MediQAl: A French Medical Question Answering Dataset for Knowledge and Reasoning Evaluation

**DOI:** 10.1038/s41597-026-06680-y

**Published:** 2026-02-05

**Authors:** Adrien Bazoge

**Affiliations:** 1 Nantes Université, CHU Nantes, Pôle Hospitalo-Universitaire 11: Santé Publique, Clinique des données, INSERM, CIC 1413, F-44000 Nantes, France; 2 Nantes Université, Ecole Centrale Nantes, CNRS, LS2N, UMR 6004, F-44000 Nantes, France

**Keywords:** Information technology, Scientific data

## Abstract

This work introduces MediQAl, a French medical question answering dataset designed to evaluate the capabilities of language models in factual medical recall and reasoning over real-world clinical scenarios. MediQAl contains 32,603 questions sourced from French medical examinations across 41 medical subjects. The dataset includes three tasks: (i) Multiple-Choice Question with Unique answer, (ii) Multiple-Choice Question with Multiple answer, and (iii) Open-Ended Question with Short-Answer. Each question is labeled as *Understanding* or *Reasoning*, enabling a detailed analysis of models’ cognitive capabilities. We validate the MediQAl dataset through extensive evaluation with 14 large language models, including recent reasoning-augmented models, and observe a significant performance gap between factual recall and reasoning tasks. Our evaluation provides a comprehensive benchmark for assessing language models’ performance on French medical question answering, addressing a crucial gap in multilingual resources for the medical domain.

## Background & Summary

Medical licensing examinations, originally designed to assess students’ knowledge and reasoning, are increasingly repurposed as benchmarks for evaluating large language models (LLMs) medical capabilities^1^. Benchmarks of question-answering tasks predominantly rely on multiple-choice questions (MCQs) with a single correct answer^2,3^. This format is widely used due to the availability of automatic evaluation metrics that provide consistent and objective assessment of LLMs at scale.

While MCQ-based datasets and metrics provide a valuable initial insight into LLM performance, they are often limited in several key aspects: the number of examples, the diversity of medical subjects covered, their representation of real-world clinical scenarios^4^, and the range of languages represented. Indeed, most of existing benchmarks are heavily centered around English, which restricts their applicability to multilingual or non-English contexts^1^. Furthermore, medical benchmarks inherently reflect cultural, educational, and regulatory contexts in which they are developed. The format of questions and answers mirrors how medicine is taught and assessed in their respective countries, which differs in structure, emphasis, and evaluative expectations across regions. In addition, treatment guidelines, clinical protocols, and legal standards are often country-specific, meaning that identical questions translated across languages can pose entirely different challenges. In this regard, French medical examinations illustrate how linguistic and pedagogical specificities can further shape the difficulty of medical question answering. French medical examination questions are typically characterized by long and syntactically complex sentences, frequent use of embedded clauses, and heavy reliance on negation and exception-based reasoning (e.g. “All of the following statements are true, except one. Which one?”). In addition, French clinical discourse often encodes temporal and causal relationships implicitly rather than through explicit markers, increasing the difficulty of clinical reasoning required to interpret the questions.

Multiple-choice question answering with a unique correct answer (MCQU) is a well-established task, frequently used to benchmark language models. In English, multiple high-quality datasets exist, including HEAD-QA^5^, MedQA^6^, MedMCQA^7^, PubMedQA^8^, MMLU (Medical)^2,3^. Similar efforts have emerged in other languages, with MCQ datasets in Chinese^9^, Polish^10^ and Spanish^11^. However, French remains underrepresented. FrenchMedMCQA^12^ is a dataset containing 3,105 multiple-choice questions, with both unique and multiple answers, but is limited to pharmacy topics. MedExpQA^11^ includes a French subset that is translated from Spanish and contains 622 examples. FrBMedQA^13^ is a dataset of 41,000 passage-questions automatically built from French biomedical Wikipedia articles. Open-ended question answering (OEQ) in medicine is even less common, even in English, due to the need for manual evaluation. For example, MEDQA-OPEN^14^ reformulates MedQA into an open-ended format, but for French, the only available OEQ dataset is MedFrenchmark^15^, which contains only 114 examples.

Recent efforts have focused on increasing diversity in question difficulty and covering a wider variety of medical subjects^16^, yet these benchmarks remain predominantly limited to English-language and rely on MCQs with a single correct answer. This limitation is particularly problematic, as several studies have demonstrated significant performance disparities between languages, with LLMs performing considerably better in English compared to less-resourced languages^11,17,18^. Therefore, it is crucial to develop more inclusive benchmarks that cover a broader range of languages and are more reflective of real-world clinical scenarios, ensuring a fair and comprehensive evaluation of LLMs in the medical domain.

In this work, we present MediQAl, a medical question answering dataset for French. This dataset contains questions sourced from French medical licensing examinations. These are manually created by academic and hospital faculty members to reflect real-world clinical scenarios and cover a broad range of medical subjects. This paper makes the following contributions:

1. We introduce MediQAl, a French medical question answering (QA) dataset that includes three tasks: (i) Multiple-Choice Question with Unique Answer (MCQU), (ii) Multiple-Choice Question with Multiple Answers (MCQM) and (iii) Open-Ended Question with Short-Answer (OEQ).
2. MediQAl covers a total of 41 medical subjects and each question is categorized as either *Understanding* or *Reasoning*, enabling detailed analysis of LLMs’ capabilities across different cognitive tasks.
3. We present an extensive evaluation of 14 large language models (LLMs) on MediQAl, including latest reasoning-based models, providing a comprehensive benchmark for assessing their performance over real-world clinical scenarios. We compare different groups of models, focusing on the performance gap between vanilla models (non-reasoning) and their reasoning-enhanced counterparts.

## Methods

### Data collection

For the construction of this dataset, the raw data was collected from publicly available websites and forums where professors and students share examination questions intended for training purposes in preparation for the national French medical examination, such as ECN exams. The National Classifying Tests (*Épreuves Classantes Nationales - ECN*) are the theoretical exams conducted during the sixth year of medical studies in France. These exams determine the ranking of medical students, which in turn allows them to select their university hospital for residency, as well as their specialization track and the services where they will complete six-month clinical internships. The ECN also serves as a comprehensive evaluation of the students’ medical knowledge and clinical reasoning, crucial for their future roles as medical practitioners. The exam consists of the following components: (i) clinical scenario-based questions, (ii) isolated knowledge-based questions, and (iii) critical article analysis questions. The question formats include multiple-choice questions (MCQs) with five options (either a single correct answer or multiple correct answers) and open-ended short-answer questions. Each year, examination questions and answer are manually created and verified by a scientific advisory board composed of tenured academic and hospital faculty members.

In line with this structure, we organized the dataset into multiple subsets corresponding to the ECN’s questions formats. The multiple-choice questions (MCQU and MCQM) were automatically extracted from the qcmlab website (https://qcmlab.net/revision.php) in March 2024. Each instance in these subsets contains a unique ID, an optional clinical scenario, a question, five candidates, the associated medical subject and the correct answer.

For the open-ended questions with short-answer (OEQ subset), the raw data was collected from multiple sources as HTML and PDF files. HTML files were well-structured enough to automatically extract QA instances using regular expressions. For the remaining PDF files, their structure was not homogeneous and could not be parsed automatically, and each instance was then extracted and curated manually.

### Data filtering

To ensure that the collected instances were homogeneous and that the questions were answerable, we applied several filters and preprocessing steps.

For the MCQU and MCQM subsets, 547 questions with missing correct answers or candidate options were removed. Since these subsets contained a large number of questions, we used three models (Llama-3.1-8B-Instruct^19^, Qwen2.5-7B-Instruct^20^ and Mistral-7B-Instruct-v0.3^21^) to vote on and filter questions to only keep challenging questions in the test sets. If any of the models answer a question correctly, the question is deemed too simple and is removed from the test sets. All filtered-out questions were then randomly split into training (80%) and validation (20%) sets for both MCQU and MCQM subsets. The dataset splits for all tasks are presented in Table 1.

**Table 1:** MediQAl dataset distribution.

	Train	Validation	Test
MQCU	10,113	2,561	4,343
MCQM	5,767	1,466	3,384
OEQ	—	—	4,969

For the OEQ subset, questions with clinical scenario containing images or tables were removed. Points awarded for each response element, sometimes embedded in the response text, were removed. Duplicates questions were identified by calculating cosine similarity on TF-IDF^22^ vectorized representations of both questions and answers. All QA pairs with a similarity score greater than 0.70 were manually reviewed, and duplicates were removed. To retain only short-answer questions, we tokenized each answer using a French medical tokenizer from DrBERT model^23^ and excluded instances where the length of the answer exceeded 200 tokens. All these filtering steps removed a total of 283 questions from the OEQ subset.

### Data quality assessment

The quality of the remaining questions after automatic filtering was assessed by conducting a human evaluation with a medical expert. We selected a stratified sample of 150 questions (50 from each test set) that were reviewed. The medical expert judged 124/150 questions to be correct. Nine questions were labeled as incorrect, identifying instances where the answer did not align with established medical knowledge. Seven questions were marked as outdated, meaning that while the answer was consistent with medical guidelines at the time of data creation, current recommendations have since changed. Four questions were found to lack sufficient clinical context to support a definitive answer, due to the use of isolated questions from clinical case that are sometimes multi-step. Finally, six questions were categorized as out of the expert’s medical domain and could not be reliably assessed.

### Medical subject annotation

**Fig. 1:**
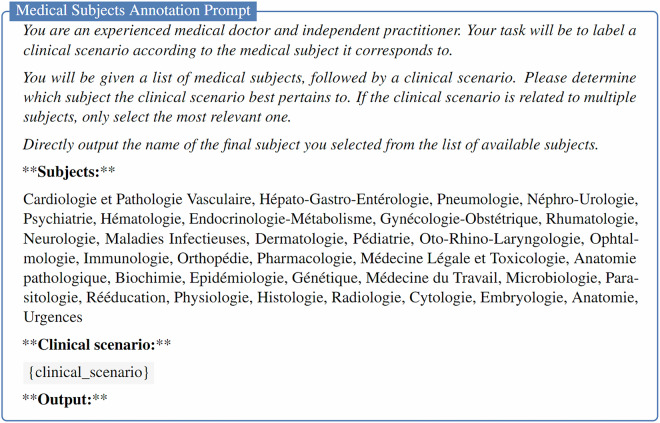
Prompt for Medical Subjects Annotation in the MediQAl-OEQ subset. The list of medical subjects in the prompt was made from the list of medical subjects from MCQU and MCQM subsets.

For MCQU and MCQM subsets, the medical subject for each question was directly available in the collected data sources. However, for the OEQ subset, the medical subjects were not consistently present across all data sources. To address this, we instructed GPT-4o (gpt-4o-2024-08-06)^24^ to automatically assign a medical subject to questions when it was missing. The prompt used for this annotation is provided in Fig. 1.

### Understanding and reasoning questions

**Fig. 2:**
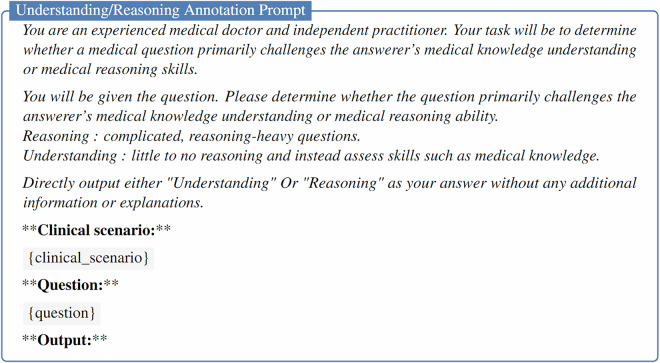
Prompt for labeling questions as *Understanding* or *Reasoning*.

To assess the capacity of LLMs to handle complex clinical reasoning tasks beyond simple recall of medical knowledge, we implemented an automatic question categorization approach. Specifically, we categorized each question into one of two types: *Understanding* or *Reasoning*. This categorization was performed using GPT-4o, following the strategy outlined by Zuo *et al*.^16^. The details of the prompt used for this process are provided in Fig. 2.

The quality of this automatic categorization was manually assessed by reviewing 10 randomly selected questions for each medical subject (5 labeled as *Understanding*, and 5 as *Reasoning*) from the test set of each task. In total, 858 questions were reviewed. Among these, 72 questions were explicitly mislabeled, resulting in an error rate of 8.4%.

## Data Records

MediQAl^25^ is available on Hugging Face (https://huggingface.co/datasets/ANR-MALADES/MediQAl) and Zenodo (10.5281/zenodo.18220039) under CC-BY-4.0 license. It consists of questions sourced from French medical examinations. MediQAl is designed to evaluate medical knowledge and reasoning on both isolated and in-context questions reflecting real-world clinical scenarios. The dataset includes three subsets, corresponding to distinct question answering tasks:

1. Multiple-Choice Question with unique answer (MCQU). This task can be formulated as *X = {C, Q, (O*_*1*_*,…, O*_*5*_*), A}* where *C* is an optional clinical scenario, *Q* is the question, *(O*_*1*_*,…, O*_*5*_*)* are five candidate options and *A* is the correct answer. For a given triplet *{C, Q, (O*_*1*_*,…, O*_*5*_*)}*, the correct answer *A* is a single option *O*_*i*_ from *(O*_*1*_*,…, O*_*5*_*)*. This task is similar to most existing MCQA datasets.
2. Multiple-Choice Question with multiple answers (MCQM). This task follows a similar formulation as MCQU: *X = {C, Q, (O_1,…, O_5), A}*. However, in MCQM, the correct answer *A* is a subset of candidate options with *|A| ≥ 2*. The answer includes multiple correct options among *(O*_*1*_*,…, O*_*5*_*)*.
3. Open-Ended Question with a short answer (OEQ). The OEQ task can be formulated as *X = {C, Q, A}* where *C* is an optional clinical scenario, *Q* is the question and *A* is a short, free-text answer. The answer length is lower than 200 tokens.

**Fig. 3:**
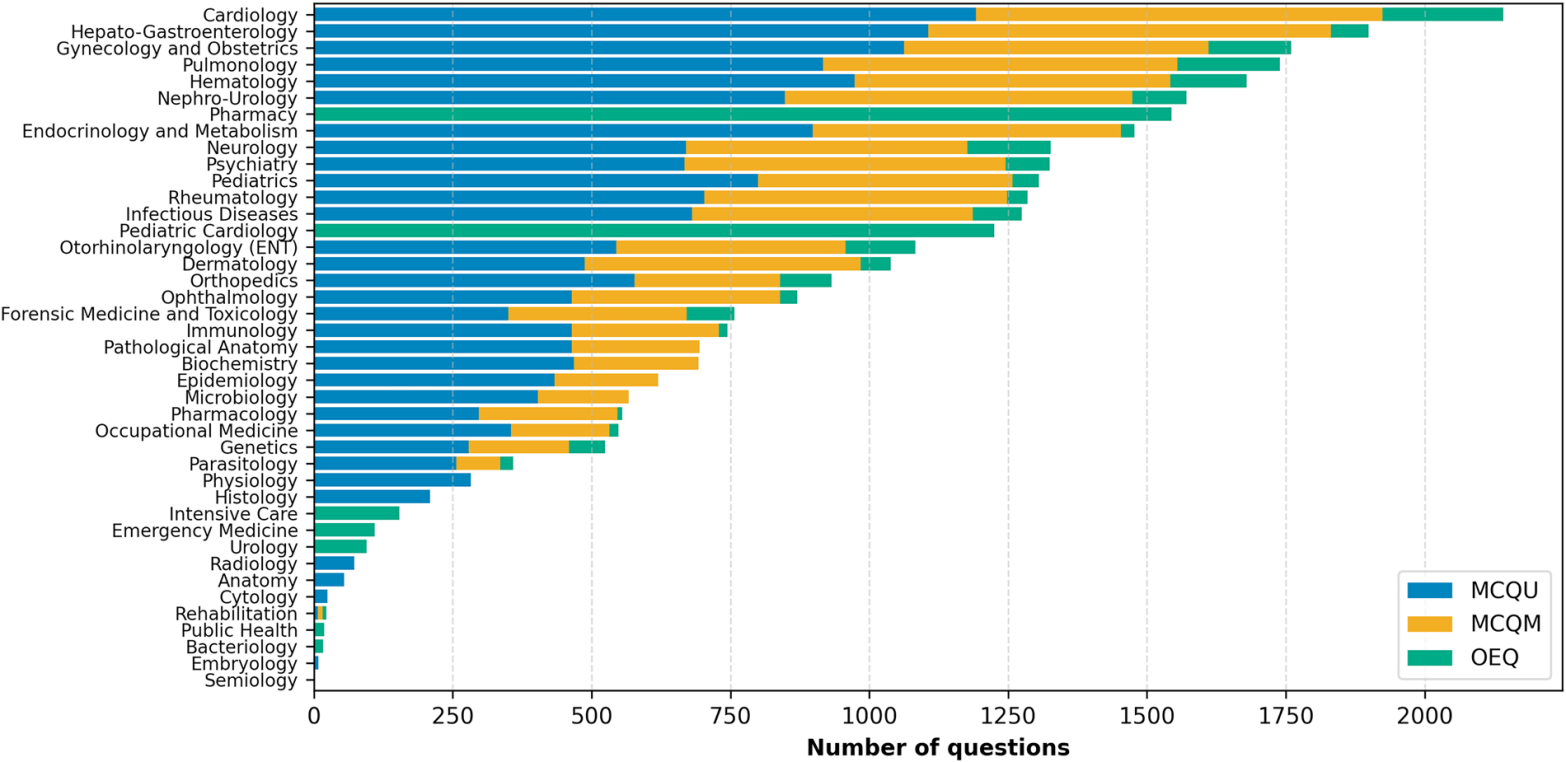
Distribution of medical subjects across MediQAl dataset.

**Fig. 4:**
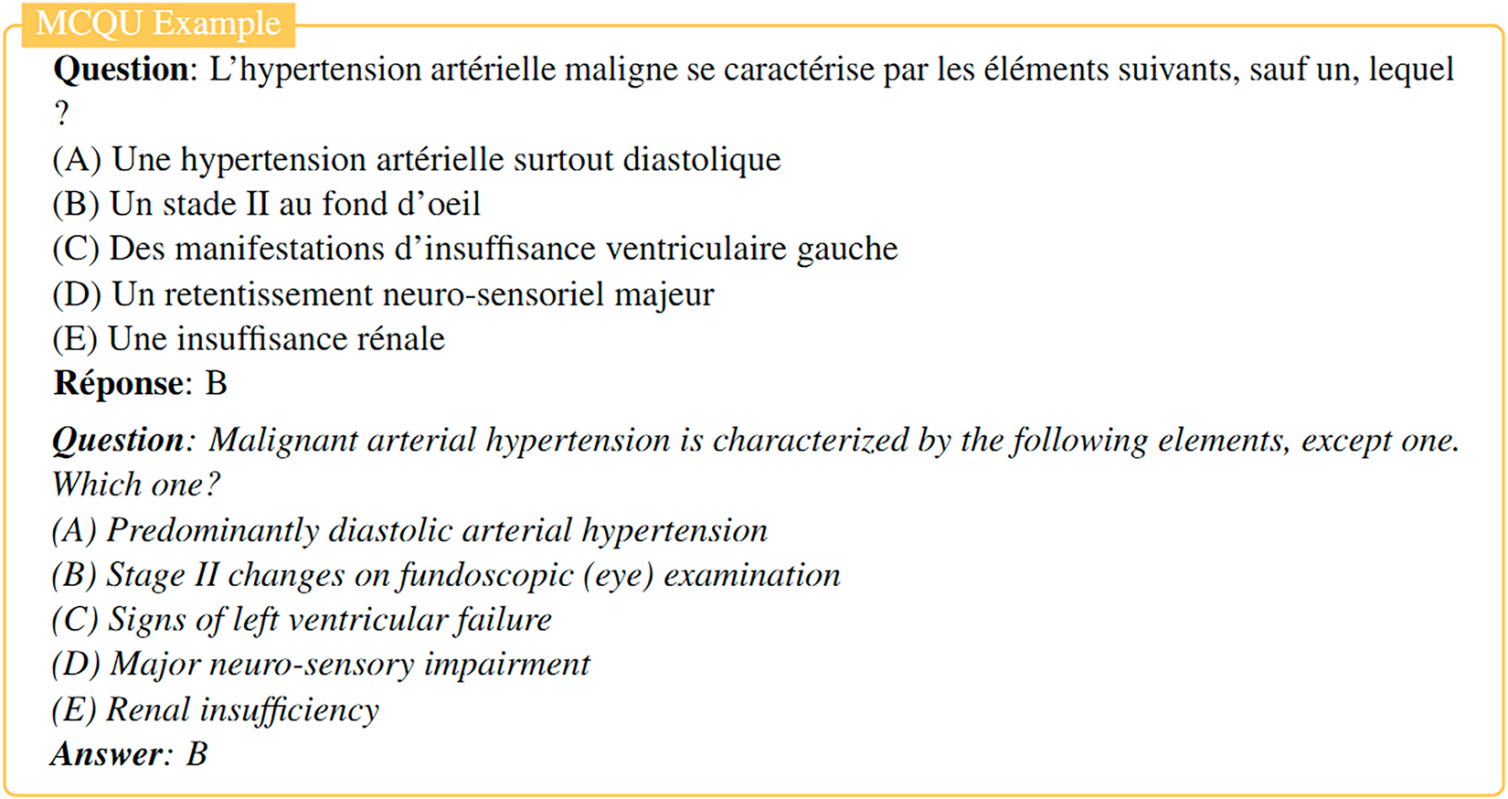
Example of Multiple-Choice Question with unique answer (MCQU) from MediQAl dataset with English translation.

**Fig. 5:**
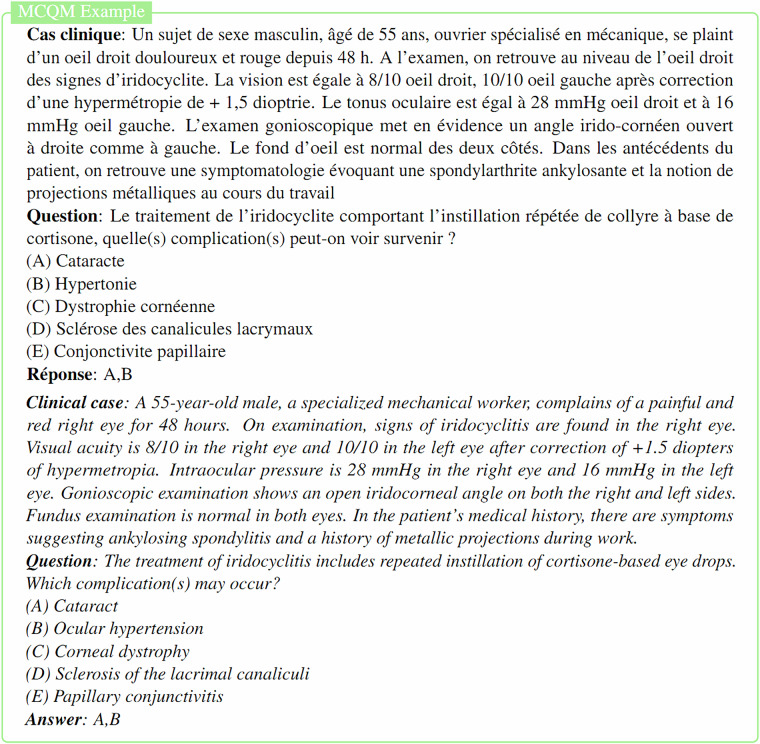
Example of Multiple-Choice Question with multiple answers (MCQM) from MediQAl dataset with English translation.

**Fig. 6:**
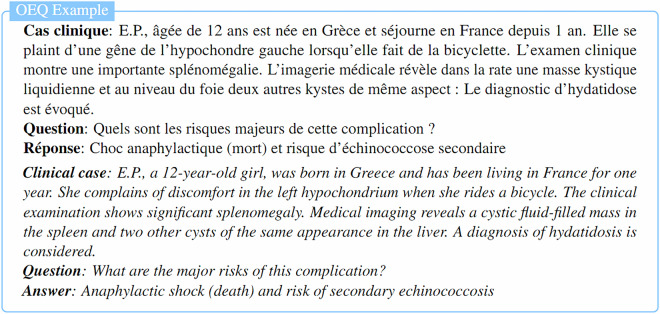
Example of Open-Ended Question with a short answer (OEQ) from MediQAl dataset with English translation.

MediQAl contains a total of 32,603 questions, of which 17,017 are MCQU, 10,617 are MCQM and 4,969 are OEQ. These questions span 41 medical subjects, as displayed in Fig. 3, and are categorized as *Understanding* or *Reasoning*, offering a diverse and reliable benchmark for medical question answering tasks in French. Table 2 summarizes the main characteristics of the dataset, and Figs. 4–6 illustrates examples for each of the three tasks.

**Table 2:** Characteristics of the MediQAl dataset. In-context questions refer to questions including a clinical scenario.

	MCQU			MCQM			OEQ		
	Understanding	Reasoning	Total	Understanding	Reasoning	Total	Understanding	Reasoning	Total
**Total number of questions**	11,336	5,681	17,017	7,742	2,875	10,617	1,842	3,125	4,969
**# Isolated questions**	9,126	961	10,087	6,200	343	6,543	836	179	1,015
**# In-context questions**	2,210	4,720	6,930	1,542	2,532	4,074	1,006	2,946	3,954
**Avg. Question length**	18.95	21.57	19.82	13.20	16.12	13.99	16.79	20.95	19.40
**Avg. clinical scenarios length**	83.50	107.67	99.97	94.87	114.77	107.24	109.71	141.28	132.19
**Avg. answer length**	—	—	—	—	—	—	25.26	40.24	34.68

Lengths are measured in words.

## Technical Validation

### Experimental setup

#### Models

We evaluate several leading LLMs on MediQAl, covering both proprietary and open-source models, including vanilla models and recent reasoning-based models. This selection ensures diversity across training paradigms, architectural families, and intended use cases, allowing us to assess how reasoning specialization and medical adaptation influence performance.

##### Vanilla large language models

GPT-4o (gpt-4o-2024-08-06)^24^, DeepSeek-V3^26^, Qwen2.5-72B-Instruct^20^, Llama-3.3-70B-Instruct^19^, Llama-3-UltraMedical 70B and 8B^27^ and BioMistral-7B^28^.

##### Reasoning large language models

o3 (o3-2025-04-16)^29^, DeepSeek-R1^30^, DeepSeek-R1-Distill-Llama-70B^30^, HuatuoGPT-o1-8B^31^, FineMedLM-o1 8B^32^, DeepSeek-R1-Distill-Llama-8B^30^, DeepSeek-R1-Distill-Qwen2.5-7B^30^.

#### Supervised fine-tuning

In addition to all evaluated models, we conducted supervised fine-tuning (SFT) on BioMistral-7B to assess the learnability and utility of the MediQAl dataset. The BioMistral-7B-SFT model was trained for two epochs using the combined training sets of all tasks (MCQU, MCQM, and OEQ). Since the OEQ subset lacks a dedicated training set, we converted questions from MQCU and MCQM training sets into OEQ format to enable unified training. We performed full fine-tuning of the model with a learning rate of 2 × 10^−5^.

#### Evaluation framework

All models across all tasks were evaluated in a zero-shot prompting setup, using greedy decoding for output generation when available to ensure result stability. For reasoning models that require specific evaluation settings, we followed the recommended instructions provided for each. To reduce inference time, open-source models were limited in their output length: up to 2,048 tokens for vanilla models and up to 8,192 tokens for reasoning-based models. For API-based models (o3, GPT-4o, DeepSeek-V3 and DeepSeek-R1), we followed the recommended prompting guidelines, removing the system prompt while keeping all other parameters at their default settings.

**Fig. 7:**
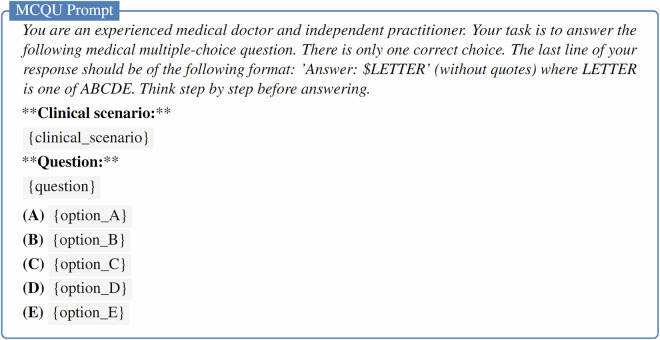
Prompt for zero-shot evaluation of LLMs on the MCQU subset. The clinical scenario is optional in the prompt.

**Fig. 8:**
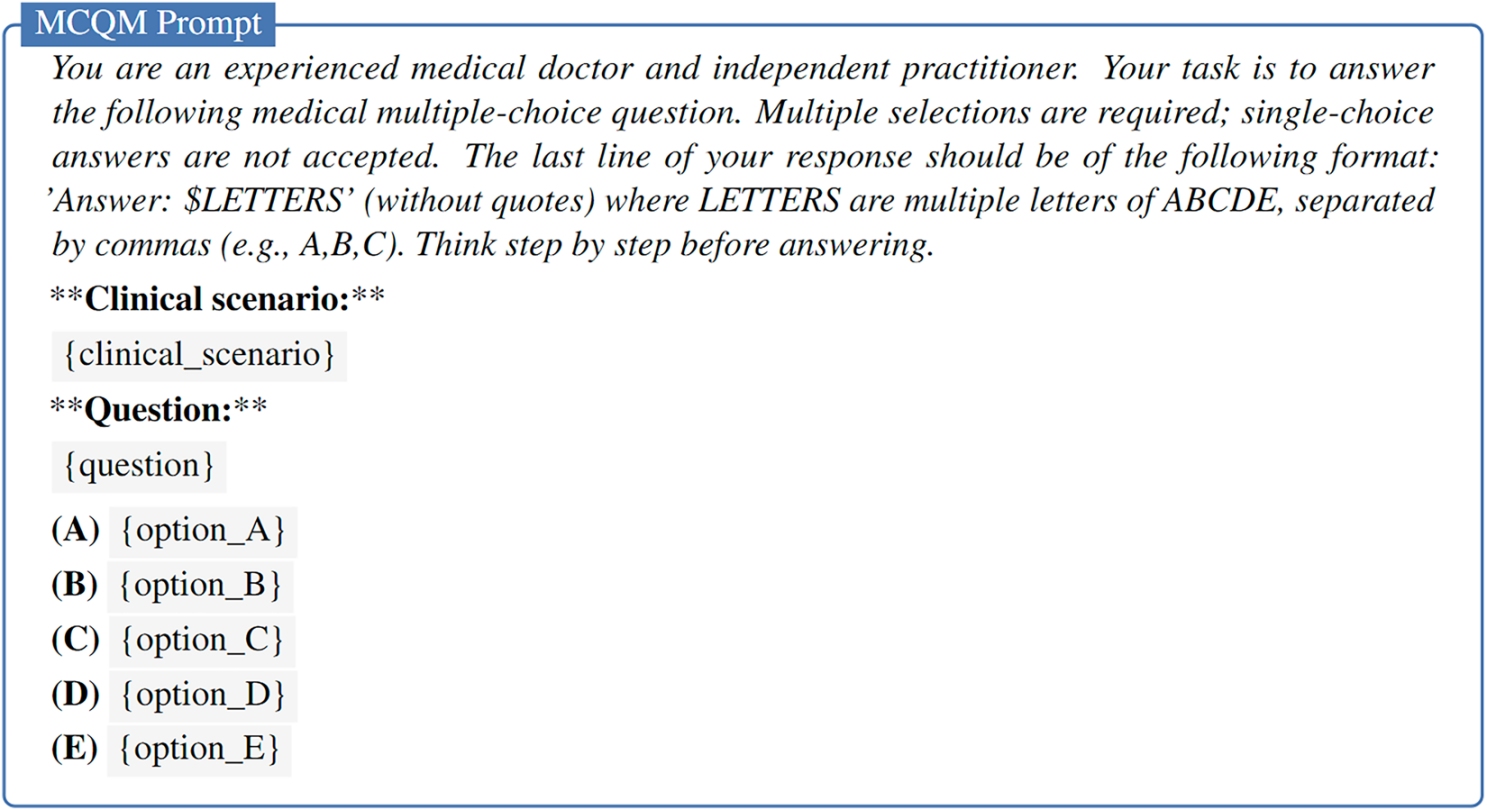
Prompt for zero-shot evaluation of LLMs on the MCQM subset. The clinical scenario is optional in the prompt.

**Fig. 9:**
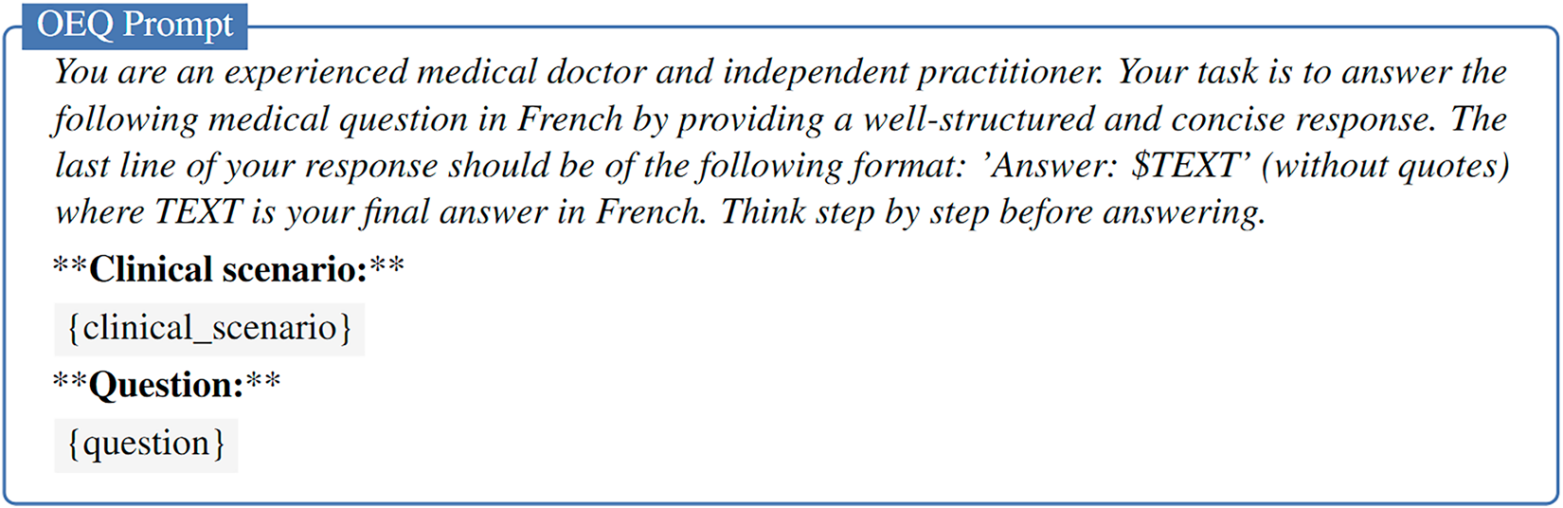
Prompt for zero-shot evaluation of LLMs on the OEQ subset. The clinical scenario is optional in the prompt.

The evaluation prompts and scripts used to extract responses from the generated text were inspired by the format of the *simple-evals* framework (https://github.com/openai/simple-evals). The specific prompts used for MCQU, MCQM and OEQ tasks are provided in Figs. 7–9, respectively.

#### Random baselines

To contextualize model performance, we included simple random baselines tailored to the structure of each task. For MCQU, we randomly selected an answer from the five available options (A to E). For MCQM, we randomly sampled one combination among all possible subsets of size two to five using the five available options (A to E). For the OEQ task, we used as answer the first L tokens of the question itself, with L set to the average answer length observed in the test set.

### Evaluation metrics

#### Multiple-choice question with unique answer (MCQU)

For the evaluation on the MCQU subset, we used Accuracy, similarly to other single-answer multiple-choice tasks such as MMLU^2^.

#### Multiple-choice question with multiple answers (MCQM)

We used Exact Match Ratio (EMR) and Hamming score to evaluate multiple-choice questions with multiple answers, following previous work on this task^12^. The two metrics are defined as follows:

$$\text{Exact Match Ratio}(\mathrm{EMR})=\frac{1}{N}\mathop{\sum }\limits_{i=1}^{N}\left[\hat{{y}_{i}}={y}_{i}\right]$$

where *N* denotes the number of questions, $\hat{{y}_{i}}$ is the set of predicted answers for the ${i}^{{th}}$ question, ${y}_{i}$ is the set of correct answers for the ${i}^{{th}}$ question, and $\left[x\right]$ is an indicator function that returns 1 if $x$ is true and 0 otherwise.

$$\text{Hamming Score}\,=\,\frac{1}{N}\mathop{\sum }\limits_{i\,=\,1}^{N}\frac{\left|{y}_{i}\cap \hat{{y}_{i}}\right|}{\left|{y}_{i}\cup \hat{{y}_{i}}\right|}$$

where $N$ denotes the number of questions, ${y}_{i}$ is the set of correct answers for the ${i}^{{th}}$ question, $\hat{{y}_{i}}$ is the set of predicted answers for the ${i}^{{th}}$ question, $\left|{y}_{i}\cap \hat{{y}_{i}}\right|$ is the intersection size between the correct and predicted answers, and $\left|{y}_{i}\cup \hat{{y}_{i}}\right|$ is the size of the union of correct and predicted answers.

#### Open-ended question with short-answer (OEQ)

To evaluate the free-text responses in the OEQ subset, we opted for a combination of lexical and contextual embedding-based metrics that align with human judgments on clinical texts^33^. These metrics are: ROUGE-1^34^, BLEU-4^35^, and BERTScore (roberta-large-mnli)^36^.

Given the inherent complexity of evaluating open-ended question answering task, where clinically acceptable responses can differ significantly in phrasing, we supplemented traditional metrics with an automatic evaluation using a LLM-as-Judge approach^37^. This strategy consists of comparing model-generated responses with expert-provided references, allowing for more nuanced assessment beyond surface-level lexical and semantic similarity. To minimize bias, we adopted Gemini-2.0-Flash^38^ as the judging model, as it was not part of the evaluated model set and does not belong to the same model family as any evaluated model.

**Fig. 10:**
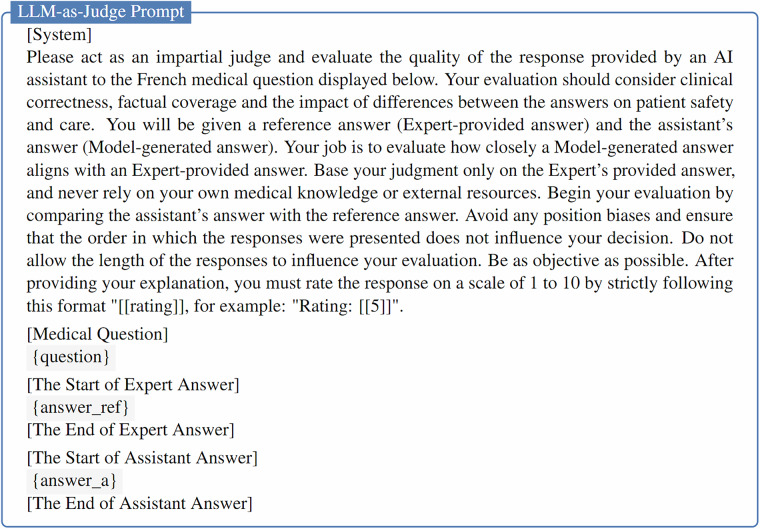
Prompt for LLM-as-judge evaluation of LLMs on the OEQ subset.

The model was prompted to assign a score from 1 to 10 for each (question, model answer, expert answer) triplet, as described in the LLM-as-Judge prompt in Fig. 10. A score of 0 was assigned to cases where the evaluated model either failed to produce a final answer or generated a response that did not conform to the expected format and was therefore unparseable. Final scores were averaged across all examples and scaled to a 0–100 range for reporting.

### Experimental results

#### Multiple-choice question with unique answer

Table 3 shows the performance of all evaluated LLMs on the MCQU subset of the MediQAl dataset. We observe that o3 achieves the highest performance on both *Understanding* and *Reasoning* questions with 73.15% accuracy. Among open-source models, DeepSeek-R1 and DeepSeek-V3 perform well on this subset with 67.03% and 63.32% accuracy, even surpassing some commercial models such as GPT-4o (60.95%). In contrast, models like DeepSeek-R1-Distill-Llama-70B, Llama-3.3-70B and Qwen2.5-72B demonstrate lower performance, correctly answering half of the questions in the test set. For smaller open-source models, HuatuoGPT-o1-8B is the only model that exceeds the random-choice baseline of 19.02% accuracy compared to others in the same size category, achieving 23.49%. Furthermore, BioMistral-7B-SFT, fine-tuned on the MediQAl training sets, shows substantial performance gains of 15.64% accuracy over its base model, BioMistral-7B. However, open-source reasoning-based models encounter difficulties due to token limitations during generation. Manual inspection of the generated text revealed that these models were still in the reasoning process after generating 8,192 tokens, resulting in incomplete answers which negatively impacts their performance.

**Table 3:** Performance of LLMs on the MediQAl-MCQU subset.

Model	Accuracy		
	Understanding	Reasoning	Average
Random baseline	20.20	18.71	19.02
*Reasoning LLMs*			
o3	**74.76**	**70.63**	**73.15**
DeepSeek-R1	69.07	63.82	67.03
DeepSeek-R1-Distill-Llama-70B	46.04	49.2	47.27
HuatuoGPT-o1-8B	24.4	22.62	23.49
FineMedLM-o1 (8B)	3.99	3.55	3.82
DeepSeek-R1-Distill-Llama-8B	9.31	6.63	8.27
DeepSeek-R1-Distill-Qwen2.5-7B	14.17	10.24	12.64
*Vanilla LLMs*			
GPT-4o	65.0	54.59	60.95
DeepSeek-V3	66.24	58.73	63.32
Qwen2.5-72B-Instruct	48.53	41.86	45.94
Llama3.3-70B-Instruct	46.57	38.31	43.36
Llama-3-70B-UltraMedical	41.52	33.27	38.31
BioMistral-7B	11.72	12.97	12.20
Llama-3.1-8B-UltraMedical	14.66	10.72	13.12
BioMistral-7B-SFT	27.81	27.89	27.84

The scores, obtained in zero-shot, are measured with Accuracy.

#### Multiple-choice question with multiple answers

Table 4 shows the performance of all evaluated LLMs on the MCQM subset of the MediQAl dataset. The best results are achieved by o3 with 55.05 EMR and 79.7 Hamming, followed closely by DeepSeek-R1 (48.88/77.54). Vanilla models such as DeepSeek-V3 and GPT-4o trail by 3–5 EMR points, indicating that additional reasoning supervision yields substantial gains. Distilled checkpoints of DeepSeek-R1 show significant performance drops (e.g. −28 EMR for DeepSeek-R1-Distill-Llama-70B, and −46 EMR for DeepSeek-R1-Distill-Llama-8B}), highlighting the trade-off imposed by aggressive model compression. In this task, open-source reasoning-based models also face the issue of still being in the reasoning phase after generating 8,192 tokens, which negatively impacts their performance and contributes to their difficulty in exceeding the random baseline score.

**Table 4:** Performance of LLMs on the MediQAl-MCQM subset.

Model	EMR			Hamming		
	Understanding	Reasoning	Avg.	Understanding	Reasoning	Avg.
Random baseline	4.76	3.8	4.46	41.64	39.21	40.89
*Reasoning LLMs*						
o3	**56.87**	**51.04**	**55.05**	**80.88**	**77.08**	**79.7**
DeepSeek-R1	51.12	43.93	48.88	79.21	73.83	77.54
DeepSeek-R1-Distill-Llama-70B	19.91	22.68	20.77	35.55	43.62	38.07
HuatuoGPT-o1-8B	8.28	5.22	7.33	47.35	41.42	45.5
FineMedLM-o1	1.46	0.19	1.06	18.76	6.93	15.07
DeepSeek-R1-Distill-Llama-8B	2.1	2.56	2.25	10.37	16.06	12.15
DeepSeek-R1-Distill-Qwen2.5-7B	2.27	2.28	2.28	19.03	19.48	19.17
*Vanilla LLMs*						
GPT-4o	46.48	37.37	43.65	76.03	69.64	74.04
DeepSeek-V3	49.18	39.37	46.13	78.45	72.02	76.45
Qwen2.5-72B-Instruct	31.8	26.47	30.14	67.38	61.5	65.55
Llama3.3-70B-Instruct	21.72	11.29	18.47	62.94	54.41	60.29
Llama-3-70B-UltraMedical	22.40	12.71	19.39	62.38	53.36	59.57
BioMistral-7B	0.82	1.33	0.98	5.33	12.41	7.54
Llama-3.1-8B-UltraMedical	5.11	4.55	4.93	44.53	40.39	43.24
BioMistral-7B-SFT	3.21	3.81	3.38	24.07	22.75	23.66

The scores, obtained in zero-shot, are measured in terms of Exact Match Ratio (EMR) and Hamming score.

#### Open-ended question with short-answer

Table 5 shows the performance of all evaluated LLMs on the OEQ subset of the MediQAl dataset. For free-text answers, the performance gap widens: o3 achieves 82.16 on the LLM-as-Judge metric, versus 74.29 for DeepSeek-R1 and 68.83 for GPT-4o. Overlap metrics (ROUGE, BLEU and BERTScore) tend to compress differences and often yield trends that diverge from those observed with the LLM-as-Judge metric. For example, DeepSeek-R1 outperforms o3 on ROUGE and BLEU scores, while the opposite is observed with the LLM-as-Judge metric. We also observed that distilled reasoning models from the DeepSeek series often reformulate the question as a multiple-choice question (MCQ) during their reasoning process, creating candidate options. This behavior poses a challenge when parsing the generated text to extract the final answer. Instead of providing a free-text response, the model tends to return the letter of one of the candidate options it created during reasoning, without necessarily including the corresponding text. This phenomenon may partly explain the comparable performance of reasoning models to vanilla models, despite their reasoning capabilities.

**Table 5:** Performance of LLMs on the MediQAl-OEQ subset.

Model	ROUGE-1			BLEU-4			BERTScore			LLM-as-Judge		
	U	R	Avg.	U	R	Avg.	U	R	Avg.	U	R	Avg.
Baseline	8.21	7.77	7.93	0.89	0.51	0.65	72.58	72.55	72.56	10.12	10.03	10.06
*Reasoning LLMs*												
o3	17.61	15.6	16.34	2.56	1.5	1.89	**77.65**	**76.48**	**76.91**	**87.4**	**79.07**	**82.16**
DeepSeek-R1	**17.8**	**15.97**	**16.65**	**2.68**	**1.67**	**2.04**	77.63	76.13	76.69	80.26	70.78	74.29
DeepSeek-R1-Distill-Llama-70B	14.41	12.57	13.26	1.85	1.43	1.58	68.64	65.3	66.54	73.16	61.3	65.7
HuatuoGPT-o1-8B	8.23	7.94	8.05	0.64	0.45	0.52	67.56	62.98	64.68	45.32	36.24	39.61
FineMedLM-o1	9.55	9.72	9.66	0.94	0.64	0.75	70.88	70.61	70.71	29.05	24.33	26.08
DeepSeek-R1-Distill-Llama-8B	5.76	5.41	5.54	0.56	0.35	0.43	60.17	55.75	57.39	21.24	16.12	18.02
DeepSeek-R1-Distill-Qwen2.5-7B	4.75	5.23	5.05	0.45	0.34	0.38	52.0	46.55	48.57	11.7	9.27	10.17
*Vanilla LLMs*												
GPT-4o	16.29	14.53	15.18	2.41	1.29	1.71	76.47	75.55	75.89	77.43	63.77	68.83
DeepSeek-V3	15.24	15.39	15.33	2.06	1.36	1.62	74.95	74.86	74.89	60.37	47.37	52.19
Qwen2.5-72B-Instruct	14.65	13.26	13.77	2.14	1.11	1.49	75.33	74.2	74.62	66.87	55.15	59.49
Llama3.3-70B-Instruct	14.53	13.59	13.94	1.7	1.02	1.27	74.56	73.49	73.89	53.32	43.77	47.31
BioMistral-7B	6.53	8.61	7.84	0.67	0.61	0.63	44.34	53.95	50.39	13.64	13.23	13.38
Llama-3.1-8B-UltraMedical	4.04	3.91	3.96	0.44	0.26	0.33	69.28	67.03	67.87	27.7	20.96	23.46
BioMistral-7B-SFT	5.69	5.59	5.63	0.6	0.38	0.47	73.75	73.53	73.61	23.86	24.38	24.19

The scores, obtained in zero-shot, are measured with ROUGE-1, BLEU-4, BERTScore and LLM-as-Judge.

#### Medical reasoning performance

Across all QA tasks, we observe a consistent performance gap between questions that require multi-step reasoning and those assessing factual recall or medical understanding. Averaged over all model-task combinations reported in Tables 3–5, accuracy on reasoning question is 5.12 points lower than understanding questions. The performance gap varies across tasks: it is largest on OEQ (7.54 points), and similar for MCQU (3.90) and MCQM (3.93). Reasoning-based models mitigate this gap to some extent but do not eliminate it. On MCQU and MCQM, the average performance gap for reasoning-augmented models is 2.15 and 2.02 points respectively, compared to 5.49 and 5.55 for vanilla models. In contrast, the OEQ task shows a large gap for both model types: 7.79 for vanilla and 7.29 for reasoning models. To illustrate, on the OEQ task, GPT-4o show a performance gap of 13.66 points between understanding and reasoning questions, which is reduced to 8.33 with its reasoning-enhanced variant, o3. A similar trend is observed for the DeepSeek family: DeepSeek-V3 shows a gap of 13.0, whereas DeepSeek-R1 narrows this to 9.48.

**Fig. 11:**
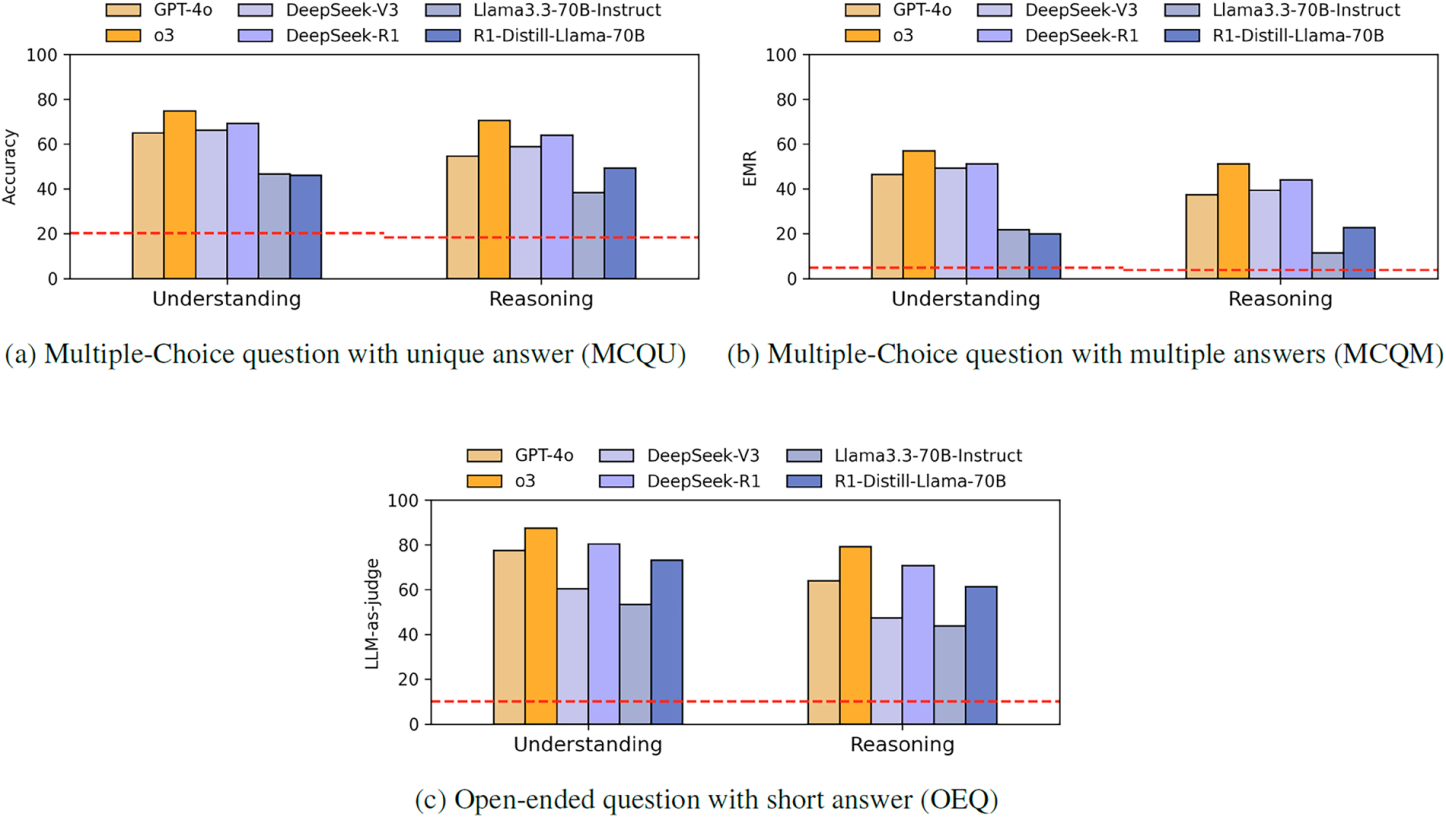
Performance of three groups of models (OpenAI, DeepSeek and Llama) on all subsets of MediQAl. The horizontal line corresponds to the random baseline.

Figure 11 presents paired performance comparisons across three model families on both *Understanding* and *Reasoning* questions:

- o3 vs. GPT-4o
- DeepSeek-R1 vs. DeepSeek-V3
- DeepSeek-R1-Distill-Llama-70B vs. Llama-3.3-70B-Instruct

Two consistent trends emerge across comparisons: (i) every model performs better on *Understanding* than on *Reasoning* questions, except for DeepSeek-R1-Distill-Llama-70B on MCQU and MQCM subsets, and (ii) when comparing each reasoning model to its base version, the performance improvement is larger on reasoning questions than on understanding questions. These differences underscore the impact of inference-time reasoning techniques. On MCQU, the average performance gain for reasoning question across the three model families is 10.67, compared to 4.02 for understanding. Similar trends are observed in MCQM, with gains of 9.87 on reasoning versus 3.51 on understanding. For the OEQ task, performance improvements are substantial in both categories with 16.57 for understanding and 18.75 for reasoning questions. These findings suggest that inference-time techniques, even without access to domain-specific adaptation, can significantly enhance complex medical reasoning. Nonetheless, even state-of-the-art LLMs remain well below human-level clinical reasoning in zero-shot settings. For downstream applications in healthcare, these models will require external verification or human oversight.

#### Medical subjects performance

To better understand the strengths and weaknesses of LLMs on our dataset, we analyzed their performance across individual medical subjects for each QA task (see Tables 6–8). In the MCQU task, the models performed best on subjects such as genetics, anatomy, dermatology, physiology, otorhinolaryngology (ENT), ophthalmology, neurology and hematology, all achieving over 80% accuracy. Conversely, subjects like cytology (notably low at 16.67% due to limited examples in the dataset), epidemiology, and psychiatry showed the lowest performance with accuracy below 60%. In the MQCM task, the easiest subjects for LLMs were dermatology, genetics, and microbiology (all above 65% EMR), while rehabilitation, occupational medicine, and pathological anatomy were the most challenging, with scores under 40% EMR. Finally, in the OEQ task, the best-performing subjects were bacteriology, parasitology, and semiology, each with LLM-as-Judge scores above 90%, whereas occupational medicine and endocrinology and metabolism were among the lowest, with score falling below 70%. These results highlight that LLMs’ capabilities vary significantly by medical domain and question type, with certain specialized or interdisciplinary fields remaining particularly challenging.

**Table 6:** Performance by medical subject on the MediQAl-MCQU subset.

Medical Subject	o3	DeepSeek-R1	DeepSeek-R1-Distill-Llama-70B	HuatuoGPT-o1-8B	FineMedLM-o1	DeepSeek-R1-Distill-Llama-8B	DeepSeek-R1-Distill-Qwen2.5-7B	GPT-4o	DeepSeek-V3	Qwen2.5-72B-Instruct	Llama3.3-70B-Instruct	Llama-3-70B-UltraMedical	BioMistral-7B	Llama-3.1-8B-UltraMedical	BioMistral-7B-SFT
Anatomy	77.78	**88.89**	33.33	33.33	0.0	16.67	33.33	77.78	83.33	50.0	38.89	50.0	22.22	11.11	22.22
Biochemistry	71.84	**73.79**	46.6	27.18	2.91	14.56	14.56	65.05	65.05	52.43	43.69	33.01	14.56	12.62	30.1
Cardiology	**78.59**	74.31	52.6	26.61	2.45	6.42	11.01	66.67	67.89	46.48	46.48	37.61	12.23	12.84	27.83
Cytology	16.67	16.67	16.67	0.0	0.0	0.0	**50.0**	16.67	16.67	16.67	16.67	16.67	16.67	0.0	16.67
Dermatology	**83.2**	73.6	50.4	16.8	4.8	5.6	14.4	70.4	76.0	51.2	52.0	38.4	14.4	13.6	41.6
Embryology	**66.67**	**66.67**	33.33	33.33	0.0	0.0	33.33	**66.67**	**66.67**	**66.67**	**66.67**	0.0	33.33	33.33	**66.67**
Endocrinology and Metabolism	**72.95**	67.15	48.31	21.74	1.93	7.25	8.21	58.94	61.84	42.51	41.06	38.65	11.59	13.53	25.6
Epidemiology	**53.61**	49.48	39.18	20.62	4.12	12.37	22.68	43.3	45.36	40.21	34.02	25.77	10.31	8.25	19.59
Forensic Medicine and Toxicology	**72.73**	47.47	43.43	20.2	3.03	10.1	15.15	53.54	51.52	36.36	35.35	27.27	11.11	11.11	21.21
Genetics	**88.89**	**88.89**	55.56	25.0	11.11	12.5	12.5	73.61	81.94	62.5	61.11	48.61	12.5	25.0	31.94
Gynecology and Obstetrics	**68.93**	62.86	45.0	14.29	1.79	5.0	8.93	52.14	55.0	44.29	41.79	40.36	10.36	12.14	28.57
Hematology	**80.76**	71.61	52.05	21.77	4.73	6.31	9.78	62.78	67.19	47.95	42.59	38.17	16.72	13.88	33.12
Hepato-Gastroenterology	**64.03**	59.71	41.01	23.38	4.32	6.12	12.23	52.88	56.47	41.01	40.29	34.17	12.23	11.51	24.1
Histology	**76.06**	60.56	43.66	16.9	4.23	14.08	15.49	63.38	59.15	46.48	45.07	30.99	8.45	16.9	18.31
Immunology	68.13	**70.33**	52.75	24.18	3.3	8.79	14.29	62.64	61.54	57.14	43.96	39.56	7.69	13.19	25.27
Infectious Diseases	**73.96**	66.15	47.92	22.4	2.6	8.33	10.94	62.5	64.58	49.48	40.62	37.5	13.02	7.81	25.52
Microbiology	**68.25**	63.49	36.51	33.33	3.17	19.05	15.87	66.67	58.73	46.03	47.62	42.86	12.7	12.7	26.98
Neurology	**80.79**	72.88	53.11	25.99	3.95	8.47	14.12	68.93	75.14	46.89	44.07	40.11	12.43	13.56	19.77
Nephro-Urology	**73.57**	71.37	48.46	30.84	2.64	5.73	9.69	64.32	68.28	48.02	43.61	41.85	11.89	11.89	27.75
Occupational Medicine	**77.57**	70.09	41.12	20.56	2.8	10.28	13.08	55.14	62.62	38.32	42.99	41.12	9.35	13.08	28.97
Ophthalmology	**77.57**	70.09	41.12	20.56	2.8	10.28	13.08	55.14	62.62	38.32	42.99	41.12	9.35	13.08	28.97
Orthopedics	**70.93**	64.53	52.33	25.58	3.49	6.98	15.7	58.72	64.53	47.67	45.93	33.72	12.79	10.47	28.49
Otorhinolaryngology (ENT)	**81.89**	75.59	55.12	25.98	3.15	8.66	11.81	73.23	72.44	50.39	47.24	44.09	9.45	17.32	37.8
Parasitology	**78.26**	63.04	43.48	28.26	8.7	6.52	13.04	65.22	63.04	45.65	50.0	52.17	4.35	17.39	23.91
Pathological Anatomy	**66.67**	61.54	37.18	25.64	6.41	7.69	14.1	53.85	53.85	47.44	35.9	37.18	14.1	24.36	15.38
Pediatrics	**69.77**	66.51	47.44	23.26	6.05	9.77	14.88	59.07	60.93	50.23	39.07	33.49	12.56	13.49	30.7
Pharmacology	**72.22**	66.67	53.7	35.19	7.41	16.67	14.81	62.96	61.11	55.56	57.41	37.04	14.81	12.96	37.04
Physiology	82.61	82.61	50.72	17.39	1.45	15.94	18.84	82.61	**84.06**	52.17	50.72	49.28	13.04	10.14	28.99
Pulmonology	**66.13**	58.87	44.76	21.77	4.84	8.06	11.29	54.44	56.85	42.74	42.34	36.69	12.9	12.5	29.44
Psychiatry	**59.86**	48.59	38.73	21.83	1.41	9.15	12.68	50.0	45.77	28.87	42.96	37.32	11.97	9.86	29.58
Radiology	**75.0**	71.43	25.0	7.14	0.0	3.57	17.86	64.29	71.43	39.29	28.57	28.57	10.71	10.71	17.86
Rheumatology	**74.64**	68.42	45.45	28.23	4.78	6.22	13.88	57.42	63.64	36.84	37.8	39.23	11.48	13.88	24.88

The scores, obtained in zero-shot, are measured with Accuracy.

**Table 7:** Performance by medical subject on the MediQAl-MCQM subset.

Medical Subject	o3	DeepSeek-R1	DeepSeek-R1-Distill-Llama-70B	HuatuoGPT-o1-8B	FineMedLM-o1	DeepSeek-R1-Distill-Llama-8B	DeepSeek-R1-Distill-Qwen2.5-7B	GPT-4o	DeepSeek-V3	Qwen2.5-72B-Instruct	Llama3.3-70B-Instruct	Llama-3-70B-UltraMedical	BioMistral-7B	Llama-3.1-8B-UltraMedical	BioMistral-7B-SFT
Biochemistry	46.67	48.89	22.22	11.11	0.0	0.0	2.22	46.67	**51.11**	42.22	20.0	22.22	0.0	0.0	0.0
Cardiology	**62.03**	54.43	27.85	8.44	1.27	2.11	2.95	49.79	48.52	29.96	18.57	23.21	0.84	5.49	5.41
Dermatology	**66.67**	58.49	27.04	4.4	0.0	2.52	4.4	47.8	54.09	35.22	17.61	16.35	1.26	3.77	3.28
Endocrinology and Metabolism	**48.89**	45.0	18.89	8.89	1.67	1.67	2.22	39.44	45.56	29.44	19.44	21.11	1.67	8.89	6.66
Epidemiology	**46.15**	38.46	21.54	10.77	1.54	1.54	10.77	35.38	44.62	23.08	15.38	27.69	1.54	9.23	2.41
Forensic Medicine and Toxicology	**47.62**	27.62	19.05	2.86	1.9	2.86	1.9	35.24	32.38	24.76	18.1	13.33	0.0	2.86	1.75
Genetics	**71.64**	67.16	29.85	13.43	1.49	2.99	4.48	61.19	65.67	41.79	32.84	29.85	1.49	7.46	5.65
Gynecology and Obstetrics	**50.27**	37.3	16.22	7.57	0.54	4.86	2.16	36.22	37.3	25.95	23.24	14.59	2.7	3.78	3.37
Hematology	**62.96**	58.33	25.93	8.8	0.0	2.31	2.31	48.15	50.93	31.94	14.35	17.59	0.46	6.48	4.35
Hepato-Gastroenterology	**50.0**	45.65	16.96	6.09	1.3	1.3	3.48	38.26	42.61	25.65	16.09	15.22	0.0	1.74	1.29
Immunology	**55.22**	53.73	23.88	10.45	1.49	2.99	1.49	46.27	50.75	35.82	34.33	29.85	0.0	5.97	2.89
Infectious Diseases	**47.14**	41.43	16.43	5.0	0.71	5.0	0.71	35.71	42.14	24.29	17.86	15.0	1.43	4.29	1.95
Microbiology	65.79	60.53	36.84	15.79	0.0	0.0	0.0	60.53	**71.05**	31.58	36.84	21.05	2.63	5.26	4.99
Nephro-Urology	**56.34**	52.58	23.0	4.69	1.88	0.94	1.88	43.19	45.54	31.92	19.25	17.37	0.0	5.16	4.55
Neurology	**63.29**	53.16	22.78	10.13	1.27	1.9	1.9	55.06	52.53	37.97	20.89	28.48	0.63	6.33	4.95
Occupational Medicine	**38.33**	28.33	8.33	6.67	0.0	1.67	5.0	30.0	31.67	18.33	6.67	10.0	0.0	1.67	0.51
Ophthalmology	**59.23**	57.69	27.69	6.92	1.54	0.77	0.0	47.69	54.62	36.92	26.15	30.0	2.31	1.54	2.12
Orthopedics	**54.37**	41.75	22.33	3.88	0.97	1.94	2.91	40.78	43.69	31.07	11.65	13.59	0.97	4.85	3.11
Otorhinolaryngology (ENT)	**62.77**	54.01	14.6	8.76	1.46	4.38	1.46	51.82	46.72	30.66	19.71	19.71	2.92	5.11	4.88
Parasitology	56.0	**64.0**	24.0	16.0	0.0	0.0	0.0	56.0	56.0	44.0	28.0	32.0	0.0	4.0	3.14
Pathological Anatomy	**43.4**	41.51	9.43	7.55	1.89	1.89	1.89	28.3	32.08	20.75	16.98	15.09	0.0	7.55	1.38
Pediatrics	**52.41**	47.59	17.93	5.52	0.69	2.76	1.38	42.07	42.07	27.59	11.03	16.55	1.38	4.14	3.88
Pharmacology	49.23	46.15	23.08	13.85	3.08	6.15	3.08	47.69	**50.77**	27.69	20.0	20.0	0.0	6.15	5.64
Pulmonology	**45.97**	43.13	14.69	4.27	0.95	0.95	0.95	34.12	39.34	27.49	14.22	15.64	1.42	6.64	6.33
Psychiatry	**47.88**	42.42	16.36	6.67	0.0	1.82	1.21	36.97	36.97	26.06	9.09	19.39	0.61	4.85	4.75
Rehabilitation	0.0	0.0	0.0	0.0	0.0	0.0	0.0	0.0	0.0	**50.0**	0.0	0.0	0.0	0.0	0.0
Rheumatology	**64.48**	60.11	21.31	7.65	1.64	1.64	1.64	55.19	56.28	34.43	24.04	21.86	0.0	4.37	2.05

The scores, obtained in zero-shot, are measured with EMR.

**Table 8:** Performance by medical subject on the MediQAl-OEQ subset.

Medical Subject	o3	DeepSeek-R1	DeepSeek-R1-Distill-Llama-70B	HuatuoGPT-o1-8B	FineMedLM-o1	DeepSeek-R1-Distill-Llama-8B	DeepSeek-R1-Distill-Qwen2.5-7B	GPT-4o	DeepSeek-V3	Qwen2.5-72B-Instruct	Llama3.3-70B-Instruct	BioMistral-7B	Llama-3.1-8B-UltraMedical	BioMistral-7B-SFT
Bacteriology	**98.82**	95.29	85.88	60.0	51.18	27.65	21.76	87.06	75.88	86.47	51.76	5.29	45.88	41.18
Cardiology	**80.74**	73.46	65.35	39.91	23.23	21.29	11.2	69.95	51.71	59.63	47.93	9.91	22.07	27.28
Dermatology	**80.74**	71.48	62.22	32.96	19.63	18.15	7.22	62.78	47.04	55.0	40.93	12.41	20.0	28.52
Emergency Medicine	**74.13**	65.05	59.82	33.49	22.94	16.88	8.35	58.53	42.48	54.04	43.49	11.83	20.92	21.93
Endocrinology and Metabolism	**66.25**	60.42	45.0	28.75	22.5	10.42	10.0	56.25	44.58	50.0	33.75	7.08	13.75	15.83
Forensic Medicine and Toxicology	**77.79**	66.63	59.53	27.44	21.28	17.56	9.53	59.19	46.28	49.77	43.37	13.49	17.21	18.02
Genetics	**85.54**	80.92	74.15	44.77	30.46	19.54	20.15	74.92	56.62	66.15	50.92	5.38	26.92	19.38
Gynecology and Obstetrics	**81.01**	70.2	64.9	43.96	23.36	20.27	12.55	68.72	48.46	62.75	51.95	16.85	26.44	26.64
Hematology	**86.42**	78.54	67.59	39.42	20.15	17.59	9.27	69.85	60.22	59.12	49.56	12.7	22.34	24.96
Hepato-Gastroenterology	**74.71**	66.32	52.94	33.82	25.15	17.21	7.21	55.88	42.06	50.15	41.03	10.0	20.29	24.85
Immunology	76.88	**78.12**	75.0	56.88	50.62	26.25	14.38	70.0	59.38	64.38	54.38	22.5	38.75	37.5
Infectious Diseases	**73.75**	70.68	57.39	37.5	22.39	14.89	8.86	62.73	44.32	53.18	40.57	11.36	17.39	22.39
Intensive Care	**76.95**	72.79	66.1	35.0	23.31	15.91	7.99	64.87	45.78	58.31	44.48	15.45	21.75	24.87
Nephro-Urology	**83.58**	75.85	66.11	42.02	23.26	18.13	10.62	70.1	54.09	59.84	48.39	11.14	24.77	26.84
Neurology	**79.2**	73.13	64.2	36.53	20.47	18.33	9.0	63.73	49.0	56.87	45.2	13.47	20.67	25.13
Occupational Medicine	**54.38**	50.0	38.75	22.5	16.88	11.25	9.38	43.75	32.5	30.0	30.62	10.62	8.75	19.38
Ophthalmology	**77.1**	69.03	56.13	41.94	23.23	19.68	6.45	60.32	46.13	62.26	40.0	11.29	21.94	25.81
Orthopedics	**77.31**	68.28	52.8	33.23	21.83	17.85	10.22	61.18	45.7	51.08	42.47	11.51	23.66	23.12
Otorhinolaryngology (ENT)	**80.95**	73.73	67.54	44.21	26.83	18.33	9.52	66.59	54.05	61.03	48.89	18.41	22.78	31.83
Parasitology	**94.35**	93.04	86.09	54.78	42.17	29.57	17.39	86.09	73.48	84.78	53.04	7.39	38.7	33.91
Pediatrics	**85.11**	74.47	53.62	34.04	23.4	18.3	9.15	65.74	43.83	52.55	45.74	9.57	18.94	23.4
Pediatric Cardiology	**81.08**	71.62	62.76	35.93	23.88	15.22	11.63	67.07	54.18	54.29	46.57	9.49	22.02	19.62
Pharmacology	68.89	**75.56**	62.22	35.56	18.89	32.22	8.89	62.22	44.44	48.89	26.67	10.0	11.11	11.11
Pharmacy	**86.51**	79.29	71.96	43.67	30.98	19.36	9.07	75.04	54.4	65.79	49.68	17.03	26.56	25.51
Psychiatry	**78.5**	69.12	62.38	36.88	21.25	22.0	9.12	63.62	46.0	60.0	47.12	14.62	20.25	30.12
Public Health	**71.05**	64.74	43.68	36.32	32.63	27.37	18.42	50.53	39.47	52.11	40.0	18.42	22.63	21.58
Pulmonology	**80.6**	72.77	61.2	43.59	25.43	18.26	9.08	65.0	48.53	59.84	47.61	17.93	19.67	30.0
Rehabilitation	**75.0**	61.67	53.33	45.0	35.0	28.33	10.0	50.0	33.33	66.67	50.0	10.0	23.33	18.33
Rheumatology	**75.14**	65.14	58.38	35.41	25.41	16.22	8.11	58.92	46.22	48.92	37.03	9.73	17.3	24.05
Semiology	**95.0**	**95.0**	85.0	60.0	15.0	5.0	10.0	85.0	65.0	65.0	50.0	0.0	60.0	10.0

The scores, obtained in zero-shot, are measured with LLM-as-judge.

To further investigate these subject-level divergences, we examined the relationship between performance and several dataset characteristics, including the number of questions per subject, average question length, and the proportion of reasoning questions. Across all tasks, performance showed little association with the number of questions per subject. However, task-dependent patterns emerged for other dataset characteristics. For MCQU, correlations between performance and both question length and reasoning proportion were negligible. In contrast, for MCQM, moderate negative correlations were observed between performance and both average question length (*ρ* = −0.28) and the proportion of reasoning questions (*ρ* = −0.38), suggesting that increased reasoning proportion contribute to subject difficulty and impact performance. This effect was most pronounced in the OEQ task, where performance across models was strongly negatively correlated with the proportion of reasoning questions (*ρ* = −0.70) and average question length (*ρ* = −0.46). Consistent with these findings, agreement in subject-wise performance rankings across models increased with task complexity, from moderate agreement in MCQU (Kendall’s W = 0.31), to slightly higher agreement in MCQM (W = 0.36), and strong agreement in OEQ (W = 0.51).

These observations suggest that subject-level performance differences arise from an interaction between subject characteristics and task-specific reasoning demands. Residual differences may also be influenced by factors not directly captured in the dataset or evaluation setup, such as variation in model architectures or differences in the composition of pre-training and fine-tuning data across models.

#### Environmental impact of models

This study required substantial computational resources, for a total of approximately 4,000 hours on A100 80GB GPUs for open source models (DeepSeek-R1-Distill, Llama3, Qwen2.5, BioMistral, FineMedLM, and HuatuoGPT). These resources were dedicated to model evaluation, experimentation with various models, and debugging. According to documentation from the Jean Zay supercomputer, the total environmental cost amounted to 1,036,000 Wh or 59,05 kgCO2eq. Additionally, the total inference cost on API for data augmentation strategies, the LLM-as-Judge evaluation, and the zero-shot evaluation of o3, GPT-4o, DeepSeek-R1, and DeepSeek-V3 on the three tasks amounted to a total environmental cost of 43.36 kgCO2eq, according to Ecologits^39^.

## Data Availability

The dataset is publicly available in JSON format on Zenodo (10.5281/zenodo.18220039)^25^ and Hugging Face (https://huggingface.co/datasets/ANR-MALADES/MediQAl).

## Code availability

All python scripts to reproduce the evaluation of models on MediQAl are available on Github (https://github.com/abazoge/MediQAl).
